# Health literacy and its associated factors among the population in two schistosomiasis-endemic villages in Jiangxi Province, China

**DOI:** 10.1097/MD.0000000000039107

**Published:** 2024-08-02

**Authors:** Kexing Liu, ChiuWan Ng, Jing Zhang, Zhaojun Li, Xiaojun Zeng, Shuying Xie

**Affiliations:** a Jiangxi Provincial Institute of Parasitic Diseases, Jiangxi Province Key Laboratory of Schistosomiasis Prevention and Control, Nanchang, China; b Centre for Epidemiology and Evidence-based Practice, Department of Social and Preventive, Medicine, Faculty of Medicine, University of Malaya, Kuala Lumpur, Malaysia.

**Keywords:** associated factor, China, health literacy, schistosomiasis

## Abstract

This cross-sectional study aimed to assess the levels of health literacy and the associated factors among the general population living in 2 schistosomiasis-endemic villages in Jiangxi Province, China. Multistage stratified random sampling was used to select participants, and a face-to-face survey was conducted from July to August 2021 to collect participants’ socio-demographic characteristics and levels of overall health literacy (HL) and its 3 subscales: health literacy of basic knowledge and concepts (HL-BKC), health literacy of behavior and lifestyle (HL-BAL), and health literacy of health-related skills (HL-HRS). The Chi-square test and logistic regression models were used to assess the association between socio-demographic characteristics and low HL levels. The prevalence rates of low overall HL, HL-BKC, HL-BAL, and HL-HRS were 84.3%, 61.8%, 82.6%, and 86%, respectively. In addition, no significant differences (*P* > .05) were noted between the 2 villages regarding overall HL scores and the 3 subscales of health literacy scores. Older age (*P* < .001), occupation (*P* < .001), lower educational level (*P* < .001), and lower annual household income (*P* < .05) were associated with an increased risk of low HL. Multivariate logistic regression revealed that occupation as a student (OR = 32.289, 95% CI:1.965–530.462, *P* < .05) and fishermen (OR = 27.902, 95%CI:1.91–407.642, *P* < .05), lower education level (OR = 0.384, 95%CI:0.149–0.99, *P* < .05), older age (OR = 5.228, 95%CI:1.458–18.75, *P* < .001), and lower annual household income (OR = 0.452, 95%CI:0.24–0.851, *P* < .05) were independently associated with low HL. The prevalence of low HL is high among the population in the schistosomiasis-endemic villages of Jiangxi Province, China. Age, education level, occupation, and annual household income were all independent factors associated with HL levels. Health educational interventions to improve HL should be simultaneously conducted in health promotion work to reduce risky habits.

## 1. Introduction

Schistosomiasis, an important and neglected public health disease, has been prevalent for a long time in China, dating back 2100 years ago. It has a significant impact on the health of people and the socioeconomic development of endemic areas. Globally, it is endemic to 78 countries and regions, and an estimated 779 million people are at risk of infection.^[1]^ The latest endemic status of schistosomiasis report shows that by the end of 2022, among the 12 endemic provinces, 452 counties were found to be endemic for schistosomiasis in China, including 27434 endemic villages covering around 73 million people at risk of infection. As 1 of the 12 endemic provinces in this country, Jiangxi Province still has 39 endemic counties, including 295 endemic villages and covering 5 million people at risk of infection.^[2]^

People are infected with schistosomiasis from contact with infected water containing cercariae, so the key to preventing schistosomiasis is to change the residents’ daily risky behavior and lifestyle. For example, fishermen go fishing barefoot in endemic areas.^[3]^ Health education focuses on changing people behavior to control the spread of the disease, which is one of the crucial measures for the active prevention of schistosomiasis. Studies conducted in the Poyang Lake area^[4]^ and Dongting Lake area^[5]^ in China have shown that the prevalence of schistosomiasis infection among students significantly decreased after implementing health education interventions that provided children with adequate knowledge of schistosomiasis transmission, changing attitudes, and behavior toward the prevention and control of schistosomiasis. A recent study conducted in the Dongting Lake Basin, China, showed that the high prevalence of schistosomiasis in this area was mainly a result of inadequate knowledge, attitude, and practice toward schistosomiasis prevention among fishermen and boatmen.^[6]^ To this end, China views health education as an effective strategy for the national schistosomiasis control program. However, self-management and health behaviors are closely related to HL, which is of great importance for effectively comprehending and using health information, health education materials, and seeking healthcare services.^[7]^

HL, the ability to seek, understand, and utilize health information, has been identified as an essential factor in the process of disease prevention and control.^[8]^ People with adequate HL can more easily understand health education materials and use health information more proactively to regulate their behavior,^[9]^ such as avoiding contact with water contaminated by cercariae or taking protective measures, such as wearing protective gloves and applying anti-larvae cream when they need to be in contact with the water.^[1,10]^

HL is a crucial determinant and outcome of health education.^[11]^ Many factors can affect an individual level of HL. Research shows that public HL decreases with increasing age.^[12,13]^ The ability to understand and distinguish information is affected by education level. People with low education levels have poorer HL.^[8,14]^ By contrast, those with a high degree of education have higher HL because of their ability to seek, understand, and use important medical professional terms and knowledge.^[15,16]^ According to the American Health Literacy Survey.^[17]^ Chaudhry found that racial differences were associated with HL among people with heart disease,^[18]^ and Keller study of adolescent women with bulimia found that religious beliefs were also associated with HL.^[19]^ Furthermore, socioeconomic and population status, culture, social support, medical environment, and education system all have a significant impact on the level of public HL.

Therefore, measuring HL, identifying vulnerable populations, and devising interventions to improve HL are all critical aspects of the implementation of a health education program. However, limited HL is a significant public health problem in China, especially in undeveloped and rural areas. Although there are numerous published studies on HL among the general population and the specific population in China, to the best of our knowledge, there is a lack of research pertaining to the levels of HL as well as its associated factors in Jiangxi Province, especially in schistosomiasis-endemic areas. Assessing the level of HL among the population in endemic villages could provide helpful information for the planning, conduct, and success of schistosomiasis prevention programs in Jiangxi Province, China.

The objectives of the study were to assess the HL levels and the association between socio-demographic factors and HL among the population in 2 villages in Jiangxi Province, China.

## 2. Materials and methods

### 2.1. Study design

This cross-sectional study aimed to assess HL levels and associated factors among residents living in 2 schistosomiasis-endemic villages in Jiangxi Province, China, using the National Residents’ Health Literacy Surveillance Rapid Assessment Questionnaire (HLSRAQ). Two villages, Lantian Village, Yujiang District, and Sishui Village, Duchang County, were randomly sampled: one from the transmission-eliminated and the other from the transmission-controlled areas in Jiangxi Province.

### 2.2. The questionnaire

The questionnaire on HL used in the study consisted of 2 sections. The first component has 9 questions about socio-demographic variables such as age, gender, educational level, occupation, annual household income and number of people in the household. The second section assesses participants’ health literacy using the HLSRAQ, which consists of 3 subscales with 19 items, including 7 items in HL-BAC and 6 items in health literacy of behavior and lifestyle (HL-BAL), and 6 items in health literacy of health literacy of health-related skills (HL-HRS). There are 2 types of questions, including single choice and multiple choice. Participants get 1 and 2 points for each single and multiple-choice question if they answer correctly. The total score ranges from 0 to 29, with higher scores indicating greater HL. Participants who scored <24 points (equivalent to 80% out of full mark) were classified as having low HL. This questionnaire has been validated for use in China, whose Cronbach α was 0.82.

### 2.3. Study population

Residents from the 2 villages comprised the study population. The inclusion criteria of this study were: living in the villages at least in the past 6 months; can read and speak Mandarin; Aged 15 to 69 years old. The exclusion criteria of this study were residents living in a dormitory, such as military bases, hospitals, prisons, and nursing homes.

### 2.4. Sampling method

**Figure 1.**
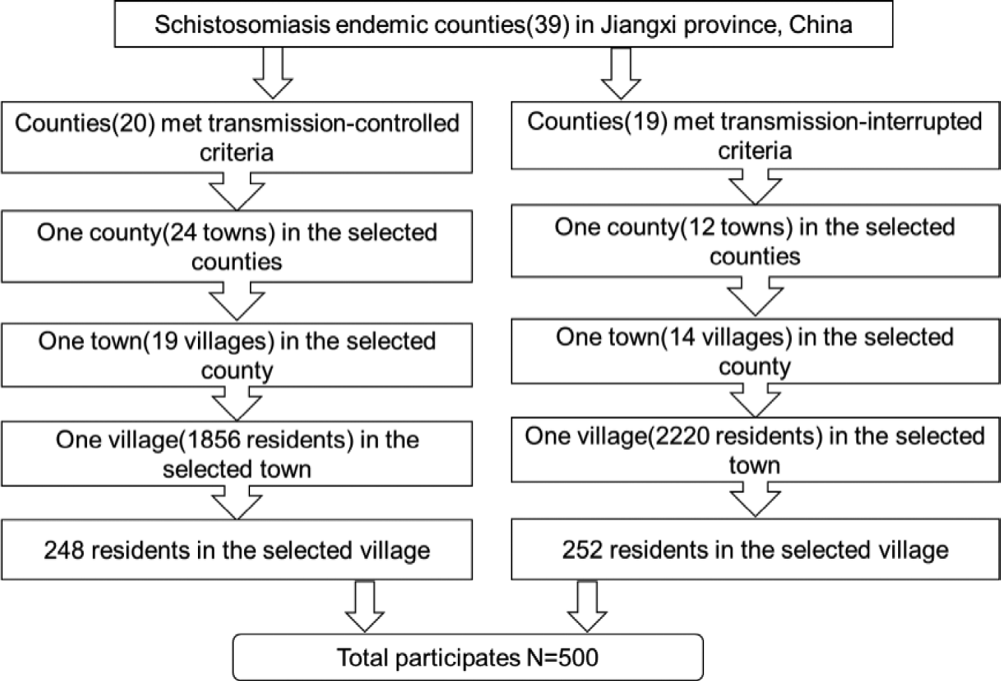
The total number of participants was selected from the 2 schistosomiasis-endemic villages. One village from countries met transmission-controlled criteria, and the another from countries met transmission-interrupted criteria. The arrows represent that the selection process is carried out in order from large to small administrative divisions (county, town, village).

In this study, 2 villages were randomly selected using a multistage random sampling method from areas that met the schistosomiasis transmission-interrupted and schistosomiasis transmission-controlled criteria. The sampling method was divided into 4 stages. Firstly, we randomly coded and selected 1 country from areas that met the schistosomiasis transmission-interrupted and schistosomiasis transmission-controlled criteria, respectively. Secondly, we randomly coded and selected 1 town from the 2 counties. Thirdly, we randomly coded and selected 1 town from the 2 towns. Fourthly, we randomly selected the participants from the villages according to the national civil system which including the information of residents. OpenEpi software was used to estimate the minimum sample size required for the study according to the data of the local civil affairs system. An overview of the sampling procedure is shown in Figure 1.

### 2.5. Data collection

The data collection was conducted from July to August 2021 in the 2 schistosomiasis-endemic villages by the team with the help of local village cadres from the local village committee. Participants completed the survey. Members of the investigation team were present if the participants needed clarification and helped fill out the forms.

### 2.6. Statistics analysis

The analysis was divided into 2 parts: descriptive and inferential. Data cleaning was performed once the data were collected and entered into IBM SPSS Statistics (Version 26). Questionnaires with incomplete answers were excluded during the data cleaning stage.

The descriptive analysis describes the data from the study. A frequency table with percentages was used to summarize the socio-demographic characteristics of the respondents. Before inferential analysis, a normal distribution test was used to check the distribution of HL scores. Chi-square tests were performed to compare HL levels in subgroups based on participant characteristics. The comparison of HL levels between subgroups in the 2 villages was performed using one-way analysis of variance. After categorizing the dependent variables, univariate and multivariate logistic regressions were used to identify independent variables influencing the degree of HL among people in the 2 villages. A significance threshold of 0.05 and 0.10 is necessary for the variable selection phase to allow variables to enter and remain in the model. The Hosmer-Leme test is a goodness-of-fit test for logistic regressions. With *P* value > .05 in this model, it showed a good model fit. All analyses were conducted using SPSS software. Missing values for independent variables were calculated using multiple imputations. Hypothesis testing was performed at a significance level of 0.05.

### 2.7. Ethical consideration

Prior to conducting this study, ethical approval was obtained from the Medical Research Ethics Committee of the Jiangxi Provincial Institute of Parasitic Diseases.

Before participating in this study, all volunteers were informed of the study aims and objectives, and informed consent was obtained during the introduction. Everyone who completed the initial survey was offered a wash basin and a towel as a gift, and all the participants’ information gathered from this study was kept confidential.

## 3. Results

### 3.1. Description of participants’ characteristics

We collected 552 questionnaires between July and August 2021. A total of 534 valid questionnaires were included in the analysis; 18 questionnaires were excluded because they filled out the questionnaire twice (n = 9), were not in the selected age range (n = 6), and did not live in the villages chosen (n = 3).

The demographic characteristics of the study population are presented in Table 1. Among the 534 participants in this study, 249 people and 285 people were from Sishui and Lantian villages, respectively. A total of 228 participants (42.7%) were female, and 306 (57.3%) were male. Most participants were 55 to 64 and 65 to 69 years old, accounting for nearly 50% of the participants. Regarding educational attainment, most participants (68.2%) were educated at the illiterate, primary school, and junior school levels. In addition, farmers made up about 71.2% of the participants, while participants with 3–5 persons in the household made up 62.9%. In terms of income, 53.7% of the participants had an annual household income of 50,000 to 100,000 Chinese Yuan (CNY), accounting for the largest proportion of the participants, followed by 30.9% of participants with an annual household income of 30,000–50,000 CNY.

**Table 1:** Socio-demographic characteristics of participants in Sishui and Lantian villages.

Items	Sishui	Lantian			Total	
	Frequency (No.)	Percent (%)	Frequency (No.)	Percent (%)	Frequency (No.)	Percent (%)
Gender						
Male	140	56.2	166	58.2	306	57.3
Female	109	43.8	119	41.8	228	42.7
Age						
15–24	34	13.7	28	9.8	62	11.6
25–34	35	14.1	29	10.2	64	12.0
35–44	30	12.0	40	14.0	70	13.1
45–54	43	17.3	55	19.3	98	18.4
55–64	65	26.1	96	33.7	161	30.1
65–69	42	16.9	37	13.0	79	14.8
Ethnicity						
Han	249	100.0	285	100.0	534	100.0
Other	0	0.0	0	0.0	0	0.0
Education						
Illiterate	51	20.5	54	18.9	105	19.7
Primary school	50	20.1	59	20.7	109	20.4
Junior school	62	24.9	88	30.9	150	28.1
High school	42	16.9	32	11.2	74	13.9
College	31	12.4	38	13.3	69	12.9
Master degree or higher	13	5.2	14	4.9	27	5.1
Occupation						
Civil servant	2	0.8	2	0.7	4	0.7
Teacher	2	0.8	1	0.4	3	0.6
Medical staff	3	1.2	1	0.4	4	0.7
Staff at other public institutions	12	4.8	14	4.9	26	4.9
Student	26	10.4	21	7.4	47	8.8
Farmer	170	68.3	210	73.7	380	71.2
Worker	2	0.8	8	2.8	10	1.9
Staff at other enterprises	10	4.0	11	3.9	21	3.9
Other	22	8.8	17	6.0	39	7.3
Number of people in the household						
1–3	77	30.9	75	26.3	152	28.5
4–6	154	61.8	182	63.9	336	62.9
≥7	18	7.2	28	9.8	46	8.6
Annual household income (CNY)						
<30000	76	30.5	89	31.2	165	30.9
30000–50000	30	12.0	15	5.3	45	8.4
50000–100000	124	49.8	163	57.2	287	53.7
100000–300000	12	4.8	10	3.5	22	4.1
≥300000	7	2.8	8	2.8	15	2.8
Total	249	100.0	285	100.0	534	100.0

CNY = Chinese Yuan

**Figure 2.**
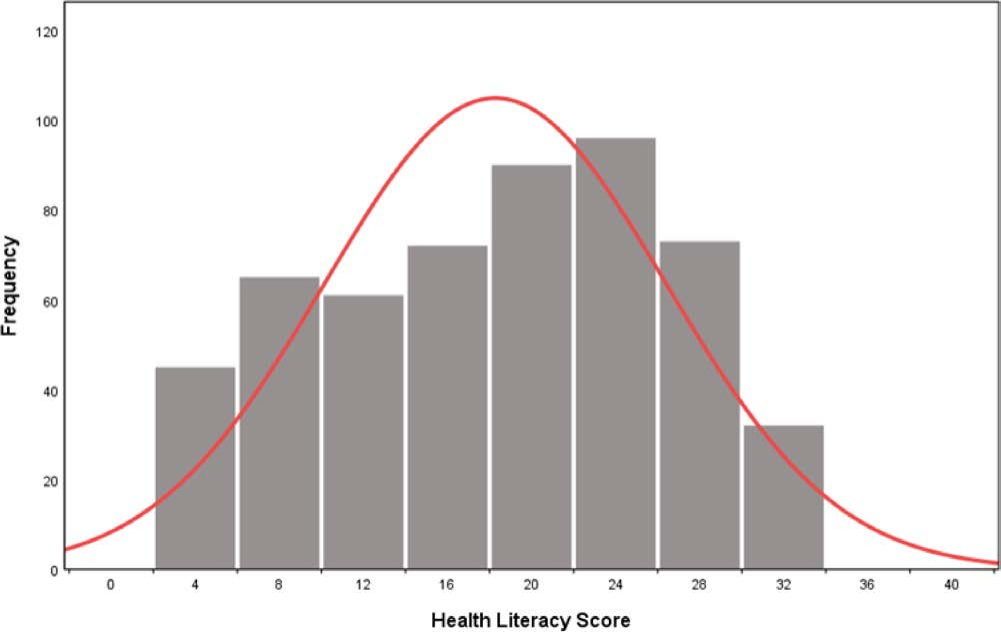
In general, the score distribution is that the middle score segment has the largest number of people, and the low and high score segments have fewer people. The distribution conforms to the normal distribution curve.

The distribution of HL scores of all participants, with the trend of increasing first and then decreasing, is shown in Figure 2. In detail, 45 (8.4%), 65 (12.2%), 61 (11.4%), 72 (13.5%), 92 (16.9%), 96 (18%), 73 (13.7%), and 32 (6%) participants had the HL score of 0 to 4, 5 to 8, 9 to 12, 13 to 16, 17 to 20, 21 to 24, 25 to 28, and 29 to 32, respectively. At the same time, the mean total HL score was 16.65 ± 8.24.

**Figure 3.**
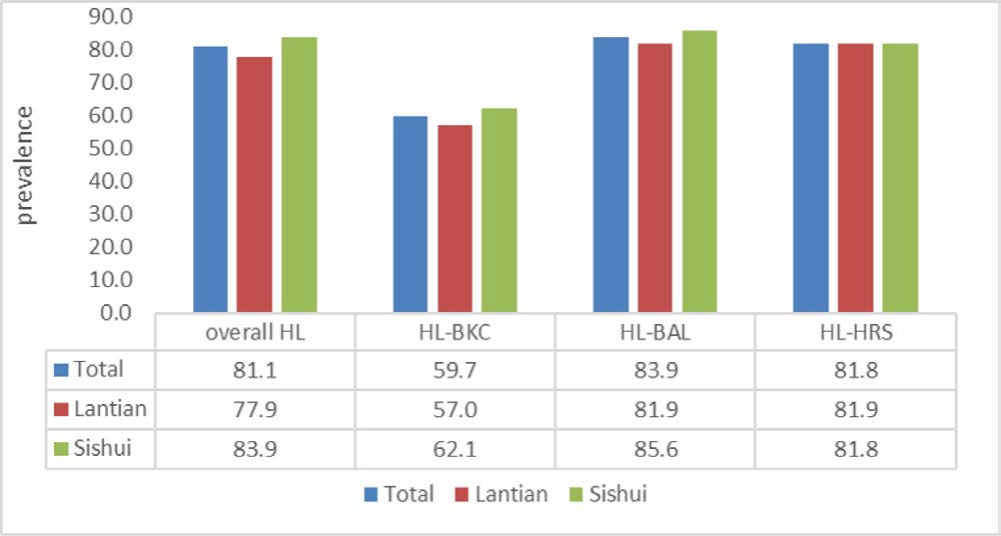
The prevalence of low overall health literacy among the participants is 81.1%. The prevalence of low health literacy regarding of overall, basic knowledge and concepts and behavior and lifestyle among participants in Sishui is higher than Lantian, expect for health-related skills.

As shown in Figure 3, the prevalence of a low level of overall HL was 81.1% (95%CI:79.3%–83.4%) among all participants, and HL-BAL had the highest level of prevalence with 83.9% (95%CI:81.2%–86.6%) in the 3 subscales. Comparing the 2 villages, Sishui had a higher prevalence rate than Lantian in overall HL, health literacy of basic knowledge and concepts (HL-BKC), and HL-BAL, except for the almost identical prevalence rate in HL-HRS.

### 3.2. Comparison of HL scores between 2 villages

Regarding the HL score, Table 2 shows that the *P* values were not significant, and there was no difference in the total HL scores or the scores of the 3 subscales in the 2 villages.

**Table 2:** Comparison of health literacy scores between the 2 villages.

Items	Location	N	Mean	Std. Deviation	t	*P*
Health literacy of basic knowledge and concepts score	Lantian	249	7.5422	3.6971	1.052	.293
	Sishui	285	7.2	3.79195		
Health literacy of behavior and lifestyle score	Lantian	249	5.1687	3.14753	0.708	.479
	Sishui	285	4.9754	3.14823		
Health literacy of health-related skills score	Lantian	249	4.2249	2.61309	−0.09	.928
	Sishui	285	4.2456	2.67145		
Overall health literacy score	Lantian	249	17.0562	8.16592	1.055	.292
	Sishui	285	16.3018	8.30748		

### 3.3. Prevalence of low levels of overall HL and its 3 subscales by socio-demographic factors

The prevalence of low health according to socio-demographic factors is shown in Table 3. Regarding the overall HL level, 83% of males and 78.5% of females had a low overall HL level. In addition, the older group had a higher prevalence of low HL (91.3% in the 55–64 age group and 86.1% in the 64–69 age group). Regarding educational level, the illiterate group had the highest prevalence of low HL (88.6%). In terms of occupation, the other occupation (fishermen) has the highest prevalence of low HL (87.2%), followed by farmers (84.7%). More than 7 participants had the highest prevalence of low HL (84.8%). In addition, people with the lowest annual household income had the highest prevalence of low HL. In the subscales of HL-BKC, HL-BAL, and HL-HRS, there was the same tendency as overall HL in different socio-demographic factors.

**Table 3:** Correlation of participants’ characteristics with low levels of overall HL, HL-BKC, HL-BAL, and HL-HRS.

Variables	Low Overall HL			Low HL-BKC			Low HL-BAL			Low HL-HRS		
	N	Rate (%)	*P*	N	Rate (%)	*P*	N	Rate (%)	*P*	N	Rate (%)	*P*
Gender			.189			.199			.085			.587
Male	254	83.0		190	62.1		258	84.3		259	84.6	
Female	179	78.5		129	56.6		179	78.5		189	82.9	
Age			.001			.001			.058			.001
15–24	44	71		16	25.8		47	75.8		47	75.8	
25–34	46	71.9		24	37.5		53	82.8		44	68.8	
35–44	54	77.1		37	52.9		56	80.0		57	81.4	
45–54	74	75.5		62	63.3		76	77.6		81	82.7	
55–64	147	91.3		123	76.4		141	87.6		152	94.4	
65–69	68	86.1		57	72.2		64	81.0		67	84.8	
Education			.001			.001			.368			.001
Illiterate	93	88.6		83	79.0		84	80.0		96	91.4	
Primary school	91	83.5		78	71.6		90	82.6		93	85.3	
Junior school	132	88.0		100	66.7		129	86.0		141	94.0	
High school	56	75.7		33	44.6		56	75.7		58	78.4	
College	46	66.7		19	27.5		54	78.3		44	63.8	
Master degree or higher	15	55.6		6	22.2		24	88.9		16	59.3	
Occupation			.001			.001			.098			.001
Civil servant	1	25.0		1	25.0		2	50.0		1	25.0	
Teacher	2	66.7		1	33.3		1	33.3		3	100.0	
Medical staff	3	75.0		2	50.0		4	100.0		3	75.0	
Staff at other public institutions	18	69.2		10	38.5		24	92.3		18	69.2	
Student	35	74.5		12	25.5		37	78.7		37	78.7	
Farmer	322	84.7		267	70.3		314	82.6		335	88.2	
Worker	7	70.0		7	70.0		7	70.0		7	70.0	
Staff at other enterprises	11	52.4		5	23.8		15	71.4		11	52.4	
Other	34	87.2		14	35.9		33	84.6			84.6	
Number of people in the household			.637			.675			.075	33		.181
1–3	120	78.9		92	60.5		119	78.3		126	82.9	
4–6	274	81.5		197	58.6		283	84.2		279	83.0	
≥7	39	84.8		30	65.2		35	76.1		43	93.5	
Annual household income (CNY)			.003			.001			.006			.001
<30000	147	89.1		107	64.8		143	86.7		153	92.7	
30000–50000	30	66.7		11	24.4		38	84.4		30	66.7	
50000–100000	230	80.1		183	63.8		233	81.2		236	82.2	
100000–300000	16	72.7		13	59.1		12	54.5		18	81.8	
≥300000	10	66.7		5	33.3		11	73.3		11	73.3	

CNY = Chinese Yuan, HL = health literacy, HL-BAL = health literacy of behavior and lifestyle, HL-BKC = health literacy of basic knowledge and concepts, HL-HRS = health literacy of health-related skills.

### 3.4. Association between socio-demographic factors and a low level of HL

Variables with statistical significance in the Chi-square test were examined using multivariate logistic regression analysis (Table 3). As shown in Table 4, compared to 15 to 24 years old, participants age 55 to 64 (OR = 5.228, 95%CI:1.458–18.75, *P* < .01) were more likely to have a low level of overall HL. Regarding educational attainment, the participants whose educational level was high school (OR = 0.384, 95%CI:0.149–0.99, *P* < .05), college (OR = 0.259, 95%CI:0.076–0.884, *P* < .05), and master degree or higher (OR = 0.113, 95%CI:0.027–0.462, *P* < .05) were less likely to have a low level of overall HL compared to participants whose educational level was illiterate. In addition, compared to participants working as civil servants (OR = 32.289, 95%CI:1.965–530.462, *P* < .05), participants working as students and other workers (OR = 27.902, 95%CI:1.91–407.642, *P* < .05) were more likely to have low levels of overall HL. Participants with an annual household income between 50,000 and 100000 CNY (OR = 0.452, 95%CI:0.24–0.851, *P* < .05) and 100000 to 300000 CNY (OR = 0.233, 95%CI:0.073–0.748, *P* < .05) were less likely to have a low level of overall HL than participants with an annual household income <30,000 CNY.

**Table 4:** Logistic regression model analyses of factors affecting low health literacy risk.

Variables	Univariate analysis			Multivariate analysis		
	OR	OR 95% C.I.	*P*	OR	OR 95% C.I.	*P*
Age						
15–24			*Ref*			*Ref*
25–34	1.045	0.483–2.265	.91	2.329	0.75–7.238	.144
35–44	1.381	0.631–3.019	.419	1.862	0.565–6.138	.307
45–54	1.261	0.616–2.581	.525	1.363	0.401–4.628	.62
55–64	4.295	1.978–9.327	.001	5.228	1.458–18.75	.011
65–69	2.529	1.091–5.861	.031	2.692	0.706–10.268	.147
Education						
Illiterate			*Ref*			*Ref*
Primary school	0.652	0.297–1.431	.286	0.759	0.336–1.712	.506
Junior school	0.946	0.435–2.058	.889	1.269	0.557–2.89	.57
High school	0.401	0.18–0.895	.026	0.384	0.149–0.99	.048
College	0.258	0.118–0.564	.001	0.259	0.076–0.884	.031
Master degree or higher	0.161	0.061–0.425	.001	0.113	0.027–0.462	.002
Occupation						
Civil servant			*Ref*			*Ref*
Teacher	6.258	0.221–162.531	.287	21.842	0.612–779.538	.091
Medical staff	9.161	0.367–220.927	.178	13.182	0.411–422.265	.145
Staff at other public institutions	6.750	0.605–75.27	.121	11.881	0.856–164.979	.065
Student	8.750	0.829–92.322	.071	32.289	1.965–530.462	.015
Farmer	16.655	1.703–162.893	.016	6.916	0.48–99.697	.155
Worker	7.529	0.501–97.751	.148	4.141	0.209–81.972	.351
Staff at other enterprises	3.355	0.294–37.103	.334	5.496	0.379–79.679	.212
Other	20.463	1.76–236.437	.016	27.902	1.91–407.642	.015
Annual household income (CNY)						
<30000			*Ref*			*Ref*
30000–50000	0.245	0.111–0.539	.001	0.433	0.17–1.101	.079
50000–100000	0.494	0.28–0.873	.015	0.452	0.24–0.851	.014
100000–300000	0.327	0.113–0.941	.038	0.233	0.073–0.748	.014
≥300000	0.245	0.075–0.797	.019	0.569	0.143–2.26	.423

CI = confidence interval, CNY = Chinese Yuan, OR = odd ratio, Ref = reference.

## 4. Discussion

### 4.1. HL levels

This study discovered that the prevalence of low HL among the population living in schistosomiasis-endemic counties of Jiangxi Province, China, was 81.1%, which is lower than the 82.39% reported in Shishou City, Hubei Province in 2017,^[20]^ 83.1% in Xingtai City, Hebei Province in 2019,^[21]^ and 81.4% in Anhui Province in 2018.^[22]^ The decrease in prevalence might be a result of health knowledge dissemination and health behavior education regarding COVID-19 in the last 2 years.^[23]^ However, compared with the national average of 78.99% reported in 2020 and other developed areas, such as 80.7% in Wuhan City, Hubei Province reported in 2018,^[24]^ and 72% in Beijing reported in 2015,^[25]^ there is still a large gap. The difference in the prevalence of low health may be due to. The economic level of Jiangxi Province is lower than the national economic level of Hubei Province and Beijing City.^[26]^ Moreover, our study was conducted in rural areas, where the financial level was significantly lower than the city average level.

In terms of the 3 subscales, the prevalence of low HL-BAL (83.9%) and HL-BKC (59.7%) were highest and lowest in the 3 subscales, respectively. This study suggests that residents may be unaware that a healthy lifestyle is beneficial for sustaining health and avoiding diseases, instead of only healing or easing their symptoms, which is consistent with earlier research.^[21,22,24]^ This phenomenon may be explained by several reasons. First, as China is still a developing country, cultural and economic constraints may exacerbate this lack of awareness. Second, sufficient HL requires a wide range of abilities; in other words, improving population HL is a difficult task that might lead to a high prevalence of residents’ low HL. Third, programs on health education by the government or public health institutions are lacking, which is a predominant cause of low HL among residents. The questions on basic knowledge and concepts were more accessible to answer than those on the other 2 HL subscales. For example, promotion of mental health regarding behavior and lifestyle, or the calculation of BMI in terms of HL-HRS, are more challenging to answer compared with questions about symptoms of fever with regard to HL-BKC.

In addition, this study was conducted only in rural areas, and we found no difference in HL levels between the 2 villages. In previous studies, residents living in a rural location were significantly associated with HL compared to those living in urban areas.^[21,24,27]^ However, our research also shows that low HL remains prevalent among rural residents. The significant proportion of low HL reveals that the challenge is not only for policymakers, academic researchers, and healthcare practitioners, but also for all inhabitants.

### 4.2. The impact factors of low HL

In this study, as age increased, people had lower levels of HL. This finding is consistent with those of previous studies conducted in other locations.^[21,25,28,29]^ This result can be explained by the fact that the age groups of 25 to 34 and 35 to 44 had the highest educational achievements among individuals aged 15 to 69. Adults aged 25 to 44 years have a remarkable ability to study and understand new knowledge and apply new things. They are better at comprehending health information from various media sources than older or younger people.^[28,30]^ In addition, as people age, their vision, hearing, and dementia may decline; at the same time, due to historical reasons, older Chinese people have few opportunities to learn to read and write, which may affect their ability to receive and process important information on improving health.^[31]^

Our study found that, except for illiterate, primary, and junior school, the level of HL increased with the increase in education level from high school level to master or higher degree level, which was in accordance with previous studies.^[28,29,31]^ This finding supports the conclusion that the 25 to 34 years old age group had greater HL in the current study because this is the potential age range in China when people can obtain a master degree or higher.

This study also found that students and fishermen had lower HL levels than those who worked in other occupations. This finding differed from other studies, where work as farmers was not associated with low HL.^[22,29,31]^ The difference in outcomes between this study and other studies might be that the students in this study consisted of primary and junior school students who still do not have enough knowledge about HL compared to high school or higher degree students. In addition, our study sites were located around Poyang Lake, where people usually live as fishermen with consistently low education levels, resulting in low HL. Identifying residents by occupation is more accessible than identifying education level or income.

Higher levels of HL have been linked to higher annual household income levels, which is consistent with our findings. People with lower annual household incomes may live under more pressure, limiting their time to receive and digest information on promoting and maintaining good health compared to families with higher annual household incomes. According to previous studies, low socioeconomic status is a significant factor contributing to low HL, which results in worse health outcomes and higher hospitalization rates.^[28–31]^ However, as annual household income grows, participants are likely to attach greater importance to daily self-health management and pursue a better quality of life. Therefore, an increase in annual household income is critical to improving the health of low-income communities. In addition, people of all ages and educational levels were affected by low HL. It primarily affects those with poor socioeconomic status. We must focus on these specific demographic segments and implement focused health education initiatives to promote HL.

This is a cross-sectional study to explore HL as well as the 3 subscales among the general population in schistosomiasis-endemic areas; however, there are some limitations to this study. First, the association between old age, low educational attainment, low annual household income, and occupations with low HL levels was assessed using data from our cross-sectional study, which was only conducted in one moment and cannot show a causative relationship. Second, annual household income was self-reported, which increased the possibility of recall bias. Longitudinal and experimental research is needed in the future to demonstrate causation and study how changes in socioeconomic status affect differences in HL. Finally, this study only focused on residents of the 2 villages, limiting the generalizability of the results.

## 5. Conclusions

Our study showed that low HL is prevalent among residents living in schistosomiasis-endemic areas of Jiangxi Province. Older people have low educational attainment, and their annual household income has a significant risk of low HL levels. In addition, students and fishermen are more likely to have low levels of HL than other occupations. It is essential for local policymakers to carry out health education programs according to the latest HL study to ensure that the objectives of the schistosomiasis prevention and control health promotion programs are reachable to the targeted groups.

## Author contributions

**Project administration:** Jing Zhang.

**Resources:** Xiaojun Zeng.

**Supervision:** ChiuWan Ng.

**Writing – original draft:** Kexing Liu.

**Writing – review & editing:** ChiuWan Ng, Shuying Xie, Zhaojun Li.
